# On the consistency of orthology relationships

**DOI:** 10.1186/s12859-016-1267-3

**Published:** 2016-11-11

**Authors:** Mark Jones, Christophe Paul, Céline Scornavacca

**Affiliations:** 1 LIRMM, CNRS, Université de Montpellier, Montpellier, France; 2 ISE-M, CNRS, IRD, EPHE, Université, Montpellier, France

**Keywords:** Orthology detection, Polynomial-time algorithms, Para-NP hardness, Inapproximability

## Abstract

**Background:**

Orthologs inference is the starting point of most comparative genomics studies, and a plethora of methods have been designed in the last decade to address this challenging task. In this paper we focus on the problems of deciding consistency with a species tree (known or not) of a *partial* set of orthology/paralogy relationships $\mathcal {C}$ on a collection of *n* genes.

**Results:**

We give the first polynomial algorithm – more precisely a *O*(*n* ^3^) time algorithm – to decide whether $\mathcal {C}$ is consistent, even when the species tree is unknown. We also investigate a biologically meaningful optimization version of these problems, in which we wish to minimize the number of duplication events; unfortunately, we show that all these optimization problems are NP-hard and are unlikely to have good polynomial time approximation algorithms.

**Conclusions:**

Our polynomial algorithm for checking consistency has been implemented in Python and is available at https://github.com/UdeM-LBIT/OrthoPara-ConstraintChecker.

## Background

Two genes from two different species are said to be *orthologous* if they derived from a single gene present in the last common ancestor of the two species via a speciation event, and *paralogous* if they were created by a duplication event [1]. Orthologs inference is the starting point of most comparative genomics studies, and is also a key instrument for functional annotation of new genomes. A plethora of methods have been designed in the last decade to address this challenging task, and can be roughly divided in two groups [2]. The first group of methods use clustering algorithms to detect homologous genes, i.e., genes sharing a common ancestry, and then reconstruct a *gene tree* describing the evolutionary history of this set of genes; orthology relationships are then deduced from this tree by comparing it with the *species tree*, i.e., the tree depicting the history of the species containing those genes, via *reconciliation algorithms* (see [3], among others, and [4] for a review of reconciliation algorithms). The second group of methods use other sources of information, e.g. sequence similarity or synteny, to directly estimate orthology relationships [5, among others]. The first set of methods are considered to be more accurate, but they require a prior knowledge of the species tree, and are very dependent on the accuracy of the gene trees. Unfortunately, the species phylogeny is not always known and gene trees can be highly inaccurate as a result of several kinds of reconstruction artifact, e.g. long-branch attraction (LBA) [6].

The second set of methods does not suffer from these drawbacks but still has an important weakness: given a set of genes *V*, the set of inferred orthology/paralogy relationships $\mathcal {C}$ for *V* may fail to be *satisfiable*, i.e., to simultaneously co-exist in any evolutionary history for *V*, or *consistent* i.e., such that all displayed triplet phylogenies are included in a species tree (formal definitions are given in the next section).

In the last years, the decision problems associated with these questions have been extensively studied, both when $\mathcal {C}$ is *full*, i.e., involves a constraint for each pair of genes in *V* [7, 8], and when it is not [9].

In [9], the authors give *O*(*n* ^3^) time algorithms to decide whether $\mathcal {C}$ is satisfiable and consistent under the assumption that the species tree is known – where *n*=|*V*|. These results hold whether $\mathcal {C}$ is a full set of constraints or not. They also showed how to decide whether $\mathcal {C}$ is satisfiable when the species tree is unknown but $\mathcal {C}$ is full (this problem was also considered in [10]).

In this paper, we extend the results of [9] by giving a *O*(*n* ^3^) time algorithm to decide whether $\mathcal {C}$ is consistent, even when the species tree is not known and $\mathcal {C}$ is not full, and show an application on real data. Thus the problems of deciding satisfiability, deciding consistency given a species tree, and deciding consistency with an unknown species tree, are all polynomial-time solvable. We also investigate an optimization version of these problems, in which we wish to minimize the number of duplication events in the evolutionary history for *V* – duplication minimization is a well-known criterion in phylogenomics [11]. Unfortunately, we show that all three problems are NP-hard, even when the maximum number of duplication events is 2, and are unlikely to have good polynomial- time approximation algorithms.

### Preliminaries

A *rooted tree* *T* with arc set *E*(*T*) and node set *V*(*T*) is a directed acyclic connected graph, in which every node has in-degree 1, except for a single node, the *root* – denoted by ROOT(*T*), of in-degree 0, and where the set of nodes in *T* with out-degree 0 – the *leaves* of *T*, denoted by *L*(*T*) – are univocally labeled. Throughout the paper, we will treat leaves in a tree as synonymous with the labels associated to them. We denote by *I*(*T*) the set *V*(*T*)∖*L*(*T*) – the *internal nodes* of *T*. If all nodes in *I*(*T*) have out-degree 2, we say that *T* is *binary*.

Given two nodes *x*,*y* in *T*, we say that *x* is an *ancestor of y in T*, and that *y* is a *descendant of x in T*, if there is a directed path from *x* to *y* in *T*. (Note that any node *x* is an ancestor and descendant of itself.) If *x* is not an ancestor of *y* and *y* is not a ancestor of *x*, we say that *x*,*y* are *separated in T*. If there is an arc from *x* to *y* in *T*, we say that *x* is the *parent of y in T* and that *y* is a *child of x in T*.

Given a node *x*, let DESC_*T*_(*x*) denote the descendants of *x* in *T*. Let CHILD_*T*_(*x*) denote the set of all children of *x* in *T*. Let LEAF_*T*_(*x*)=DESC(*x*)∩*L*(*T*), i.e. LEAF_*T*_(*x*) is the set of leaves in *T* that are descendants of *x*. Note that LEAF_*T*_(ROOT(*T*))=*L*(*T*). Given a set *A* of nodes in *T*, let LCA_*T*_(*A*) denote the *least common ancestor of A in T*, that is, the unique node *z* such that *z* is an ancestor of all *x*∈*A*, and no descendant of *z* has this property. Given two nodes *x*,*y*, we will often write LCA_*T*_(*x*,*y*) as shorthand for LCA_*T*_({*x*,*y*}). When *T* is clear from context, we will often omit “in *T*” and simply say that *x* is the ancestor of *y*,*y* is the descendant of *x*,*z* is a leaf, etc.

*Suppressing* a non-root node *x* of out-degree 1 in a tree *T* consists of removing *x* and making the unique child of *x* a new child of the parent of *x*. Given a set of leaves *L* ^′^⊆*L*(*T*), the *restriction of T to* *L* ^′^, denoted $T|_{L^{\prime }}$, is the tree derived from *T* by taking the minimum subtree of *T* spanning *L* ^′^, and suppressing all non-root nodes of out-degree 1.

A *triplet* is a rooted binary tree *T* with |*L*(*T*)|=3. Given three distinct elements *x*,*y*,*z*, we denote by *x* *y*|*z* the unique triplet *T* with *L*(*T*)={*x*,*y*,*z*} such that LCA_*T*_(*x*,*y*)≠ROOT(*T*) (or equivalently, LCA_*T*_(*x*,*y*)≠LCA_*T*_(*x*,*z*)=LCA_*T*_(*y*,*z*)). We say that a rooted tree *T* *displays* the triplet *x* *y*|*z* if *T*|_{*x*,*y*,*z*}_=*x* *y*|*z*.

Given a set of edges *E* over a set of vertices *V*, and a subset *V* ^′^⊆*V*, we define *E*[*V* ^′^]={*x* *y*:*x*,*y*∈*V* ^′^,*x* *y*∈*E*}. Given graphs *G*=(*V*,*E*) and *G* ^′^=(*V* ^′^,*E* ^′^), we say that *G* ^′^ is an *induced subgraph of G* if *V* ^′^⊆*V* and *E* ^′^=*E*[*V* ^′^], and denote *G* ^′^ by *G*[*V* ^′^]. We define $\overline {E} = \{xy: x,y \in V, xy \notin E\}$ and say that $\overline {G}=(V,\overline {E})$ is the *complement of G*. For any integer *l*≥1, a *path* *P*_*l*_ is a graph (*V*={*v*_1_,…,*v*_*l*_},*E*={*v*_*i*_ *v*_*i*+1_:1≤*i*≤*l*−1}. We note here that if a graph contains an induced *P*_4_, then its complement contains an induced *P*_4_ on the same four vertices.

**Species trees and DS-trees.** Let *Σ* denote a set of species. A *species tree S on* *Σ* is a binary rooted tree such that *L*(*T*)=*Σ*, used to depict the evolutionary history of the species in *Σ*.

Genes are said to be *homologous* if they share a common ancestor. Let *V* denote a set of homologous genes belonging to species in *Σ*. A *species assignment* of *V* is a function *s*:*V*→*Σ*, with *s*(*v*)=*a* representing the fact that gene *v* belongs to species *a*∈*Σ*. For a set *V* ^′^⊆*V*, we define *s*(*V* ^′^)={*a*∈*Σ*:∃*x*∈*V* ^′^,*s*(*x*)=*a*}, and $s_{|V^{\prime }}: V^{\prime } \rightarrow s(V^{\prime })$ such that $s_{|V^{\prime }}(v)=s(v)$ for all *v*∈*V* ^′^. A *DS-tree on V* is a pair (*T*,*ℓ*), where *T* is a binary rooted tree with leaf set *V* and *ℓ*:*I*(*T*)→{*D* *u* *p*,*S* *p* *e* *c*} is a function labeling each internal node *x* of *T* as a *speciation node* (if *ℓ*(*x*)=*S* *p* *e* *c*) or a *duplication node* (if *ℓ*(*x*)=*D* *u* *p*). DS-trees are used to depict the evolutionary history of the genes in *V*. When the function *ℓ* is clear from context, we will often omit it and speak only of a DS-tree *T*.

Given two genes *x*,*y* in *T*, we say that *x*,*y* are *orthologs with respect to T* if LCA_*T*_(*x*,*y*) is a speciation node, and *paralogs with respect to T* otherwise. Given an undirected graph *G*=(*V*,*E*), a DS-tree (*T*,*ℓ*) on *V* is a *DS-tree for G* (or *G* is an *orthology graph for T*) if for every *x*,*y*∈*V*, *x* *y*∈*E*⇔*ℓ*(LCA_*T*_(*x*,*y*))=*S* *p* *e* *c*. That is, *x* and *y* are adjacent in *G* if and only if they are orthologs with respect to *T*.

The presence of two homologous genes in the same species can be caused either by duplications or gene transfers [12]. So, in absence of gene transfers, homologous genes from the same species are necessarily paralogs. We formalize this idea in the following assumption.

#### **Assumption 1**

We assume in what follows that whenever we are given a graph *G*=(*V*,*E*) with a species assignment *s*, two vertices *x*,*y* of *G* are not adjacent if *s*(*x*)=*s*(*y*).

**Cographs** A *cograph* is a graph that can be generated from a single-vertex graph using the operations of *disjoint union* (taking the disjoint union of multiple graphs) and *series composition* (adding all possible edges between vertices of multiple graphs) [13]. This generation scheme yields a representation of a cograph in terms of *cotrees*. A cotree is a rooted tree *T*, with internal nodes labeled 0 (representing the disjoint union operation) or 1 (representing the series composition). Hence a cotree represents a graph *G*=(*V*,*E*) if *L*(*T*)=*V* and two vertices *x* and *y* of *G* are adjacent if and only if LCA_*T*_(*x*,*y*)=1. Observe that the cotree representation of a cograph is not unique. Also, while a cotree is not necessarily binary, any non-binary cotree can be transformed in linear time into a binary cotree with the same corresponding cograph. There are several characterizations of cographs. Among other characterizations, a cograph is a graph with no induced *P*_4_ [13]. Cographs can also be viewed as graphs where each connected component has diameter at most 2.

Hellmuth et al. [8] noted that all orthology graphs (i.e. graphs for which there exists a DS-tree) can be characterized as symbolic ultrametrics [14], and showed that a graph is an orthology graph if and only if it is a cograph [8, Corollary 4].

Thus we have a useful graph-theoretic framework for deciding on the existence of a DS-tree.

#### **Proposition 1**

For an undirected graph *G*=(*V*,*E*), the following are equivalent:

1. There exists a DS-tree for *G*;
2. *G* contains no induced *P*_4_, i.e. it is *P*_4_-free;
3. *G* is a cograph.

As cographs can be recognized in linear time [15, 16], deciding whether a graph has a DS-tree, i.e., if it is *satisfiable*, can be achieved within the same time complexity. Note, however, that not every DS-tree represents a possible evolutionary history for a set of genes. In particular, given a species assignment, different parts of a DS-tree may imply conflicting evolutionary histories for the species containing those genes. The concept of *consistency* makes this notion precise.

**Consistent DS-trees.** Given a DS-tree *T* on *V*, a species assignment *s*:*V*→*Σ* and a species tree *S* on *Σ*, we say that (*T*,*s*) is *consistent with S* (or *S-consistent*) if for every speciation node *z* in *T*, and distinct children *x*,*y* of *z*, LCA_*S*_(*s*(LEAF_*T*_(*x*))) and LCA_*S*_(*s*(LEAF_*T*_(*y*))) are separated in *S*. Given a graph *G*=(*V*,*E*) and the species assignment *s*, the pair (*G*,*s*) is *consistent with S* if there exists a DS-tree *T* for *G* such that (*T,s*) is consistent with *S*. We say that *G* (resp. *T*) along with the species assignment *s*, is *consistent* if there exists a species tree *S* such that (*G,s*) (resp. (*T,s*)) is consistent with *S* [9].

Given a DS-tree *T* on *V* and a species assignment *s*:*V*→*Σ*, let *t* *r*(*T*,*s*) be the set of triplets *s*(*x*)*s*(*y*)|*s*(*z*) for which the triplet *x* *y*|*z* is displayed by *T* with a speciation node as the root, and for which *s*(*x*)≠*s*(*y*).

Hernandez-Rosales et al. [7] showed that (*T*,*s*) is consistent with a species tree *S* if and only *S* displays all triplets in *t* *r*(*T*,*s*). In light of this result, Hellmuth et al. [10] gave a framework for finding the DS-tree and species tree for which the maximum number of triplets are displayed, using Integer Linear Programming. Lafond and El-Mabrouk [9] improved the result of [7] by showing that it is enough to consider only the triplets in *t* *r*(*T*,*s*) that have a speciation node as the root node and a duplication node as the other internal node. This can expressed in terms of the consistency of an orthology graph in the following way.

Given a graph *G*=(*V*,*E*) and species assignment *s*:*V*→*Σ*, define the set of triplets *P*_3_(*G*,*s*)={*s*(*x*)*s*(*y*)|*s*(*z*):*x* *z*,*z* *y*∈*E* and *x* *y*∉*E* and *s*(*x*)≠*s*(*y*)}. Note that as a consequence of Assumption 1, if *s*(*x*)*s*(*y*)|*s*(*z*)∈*P*_3_(*G*,*s*), then *s*(*z*)≠*s*(*y*) and *s*(*z*)≠*s*(*x*).

By Theorem 5 in [9], we have the following theorem (in fact, Theorem 5 in [9] only states that (*G*,*s*) is consistent if and only if there *exists* a species tree *S* which displays all triplets in *P*_3_(*G*,*s*), but their proof shows that (*G*,*s*) is indeed consistent with such an *S*):

#### **Theorem 1**

*[* 9 *]* Let *G*=(*V*,*E*) have a DS-tree and let *s*:*V*→*Σ* be a species assignment. Let *S* be a species tree on *Σ*. Then (*G*,*s*) is consistent with *S* if and only if *S* displays all triplets in *P*_3_(*G*,*s*).

Theorem 1 directly provides a polynomial time algorithm to decide whether a graph and a species assignment are consistent with a given species tree. The following proposition reformulates Theorem 1 in a convenient way:

#### **Proposition 2**

Given a graph *G*=(*V*,*E*), a species assignment *s*:*V*→*Σ*, and a species tree *S*,(*G*,*s*) is consistent with *S* if and only if the following holds:

1. *G* does not contain an induced *P*_4_;
2. Every triplet in *P*_3_(*G*,*s*) is displayed by *S*.

As both of the properties in Proposition 2 are hereditary, we also have:

#### **Corollary 1**

Given a graph *G*=(*V*,*E*), a species assignment *s* and a subset *V* ^′^⊆*V*, if (*G*,*s*) is consistent with a species tree *S* then $(G[V^{\prime }],s_{|V^{\prime }})$ is consistent with the species tree $S|_{s(V^{\prime })}$.

**Constraint graphs.** A *constraint graph* is a pair (*G*,*s*) where *G*=(*V*,*M*⊎*U*) is an edge-bicolored graph and *s* is a species assignment on *V*. A constraint graph aims at representing the partial knowledge about the orthology or paralogy relations between genes from *V*. The edges in *M* are *mandatory edges*, representing the pairs of genes *xy* for which we know that *x* and *y* are orthologs. The non-edges of *G* (i.e. the set of unordered pairs *uv* for which *u* *v*∉*M*⊎*U*) represent the pairs of genes *xy* for which we know that *x* and *y* are paralogs. The edges in *U* are *unknown edges*, for which we do not know if *x* and *y* are orthologs or paralogs. From Assumption 1, we have that *x* *y*∉*M*⊎*U* for any pair of genes *x*, *y* such that *s*(*x*)=*s*(*y*) (in absence of gene transfers, homologous genes from the same species are necessarily paralogs). Note that an orthology graph is a constraint graph where *U*=*∅*. A *sandwich* of a constraint graph (*G*,*s*), with *G*=(*V*,*M*⊎*U*), is a graph *H*=(*V*,*E*) such that *M*⊆*E*⊆*M*∪*U*.

As a gene is always associated with the species it belongs to, throughout this paper we will always present a DS-tree *T* together with a species assignment *s*. Thus we will speak of a DS-tree (*T*,*s*). Similarly, we will always present an orthology graph *G* together with its species assignment *s*, and speak of an orthology graph (*G*,*s*). A sandwich graph *G* ^′^ will be presented on its own without a species assignment, as a sandwich graph is defined relative to a constraint graph (*G*=(*V*,*M*⊎*U*),*s*), and so the species assignment *s* will always be clear from context.

## Methods

### Computing a consistent DS-tree

In this section, we describe a polynomial time algorithm for the following problem:

CONSISTENT ORTHOLOGY GRAPH SANDWICH problem

**Input:** a constraint graph (*G*,*s*), with *G*=(*V*,*M*⊎*U*) and *s*:*V*→*Σ* a species assignment;

**Output:** a sandwich graph *H* for (*G*,*s*) such that (*H*,*s*) is consistent (if any exists).

Observe that by Proposition 2, the CONSISTENT ORTHOLOGY GRAPH SANDWICH problem amounts to computing a sandwich cograph satisfying extra properties. The sandwich cograph problem is known to be polynomial time solvable [17]. Our algorithm can be seen as a combination of the sandwich cograph algorithm and the BUILD algorithm [18] for checking consistency of a set of triplets.

Let *G*=(*V*,*M*⊎*U*) be an edge-bicolored graph and for *F*⊆*U*, define the graph *G*(*F*)=(*V*,*M*∪*F*).

The first lemma proves that unknown edges between connected components of *G*(*∅*) can be removed (i.e. freezed as paralogy relations between genes).

#### **Lemma 1**

Let (*G*,*s*) be a constraint graph with *G*=(*V*,*M*⊎*U*). Let *CC* be the connected components of *G*(*∅*), and let $U_{CC} = \bigcup _{C \in CC}U[C]$. There exists a consistent sandwich graph of (*G*,*s*) if and only if there exists a consistent sandwich graph of (*G*_*CC*_=(*V*,*M*⊎*U*_*CC*_),*s*).

#### *Proof*

Suppose first that there exists a consistent sandwich graph *G* ^′^=(*V*,*E* ^′^) of (*G*,*s*) and let *S* be a species tree such that (*G* ^′^,*s*) is *S*-consistent. For every *C*∈*C* *C*, by Corollary 1 (*G* ^′^[*C*],*s*_|*C*_) is consistent with *S*|_*s*(*C*)_ and hence with *S*. Then the disjoint union *G* ^′′^ of the *G* ^′^[*C*] is a sandwich cograph of (*G*_*CC*_,*s*). Moreover we clearly have *P*_3_(*G* ^′′^,*s*)=∪_*C*_ *P*_3_(*G* ^′^[*C*],*s*_|*C*_), implying that (*G* ^′′^,*s*) is also consistent with *S*. The converse is symmetric. □

#### **Reduction Rule 1**

Let (*G*,*s*) be a constraint graph with *G*=(*V*,*M*⊎*U*). Remove from *U* every edge *xy* such that *x* and *y* belong to distinct connected components of *G*(*∅*).

**Fig. 1:**
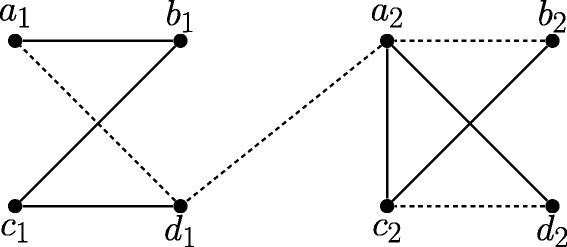
Example of an constraint graph (*G*=(*V*,*M*⊎*U*),*s*), with *s*(*a*_1_)=*s*(*a*_2_)=*A*,*s*(*b*_1_)=*s*(*b*_2_)=*B*,*s*(*c*_1_)=*s*(*c*_2_)=*C*,*s*(*d*_1_)=*s*(*d*_2_)=*D*. The mandatory edges *M* are *solid*; the unknown edges *U* are *dashed*. Reduction Rule 1 will delete the unknown edge *d*_1_ *a*_2_

As an example, consider the constraint graph (*G*,*s*) in Fig. 1. The genes *a*_1_,*b*_1_,*c*_1_,*d*_1_ form one connected component of *G*(*∅*), and *a*_2_,*b*_2_,*c*_2_,*d*_2_ form the other. Thus Reduction Rule 1 will remove the unknown edge *d*_1_ *a*_2_ from *U*.

Note that although we remove all edges between connected components of *G*(*∅*), we cannot solve the problem on each connected component independently, and so we cannot assume that *G*(*∅*) is connected. The reason is that for two connected components *C*,*D* of *G*(*∅*), a solution for (*G*[*C*],*s*_|*C*_) may be consistent with a different species tree than a solution for (*G*[*D*],*s*_|*D*_). To avoid conflicts between solutions on different subgraphs, we must split the graph into subgraphs on disjoint sets of species.

From now on, we may assume that |*s*(*V*)|>1. Otherwise, Assumption 1 implies that *M*=*U*=*∅*, and thereby (*G*,*s*) is a trivial positive instance. For the sake of the algorithm, we define an auxiliary graph *H*_*G*,*s*_=(*Σ*,*F*) on the species set, called hereafter the *species graph*. For each pair of distinct species *a*,*b*∈*Σ*, add *ab* to *F* if there exist *x*,*y*∈*V* such that *x* and *y* are in the same connected component of *G*(*∅*),*x* and *y* are not adjacent in *G*(*U*) and *s*(*x*)=*a*,*s*(*y*)=*b*.

#### **Lemma 2**

Let (*G*,*s*) be a constraint graph reduced by Reduction Rule 1. If the species graph *H*_*G*,*s*_ is connected, then (*G*,*s*) does not have a consistent sandwich graph.

#### *Proof*

Consider an arbitrary binary species tree *S*, and an arbitrary sandwich graph *G* ^′^=(*V*,*E* ^′^) of (*G*,*s*). We show that *P*_3_(*G* ^′^,*s*) contains a triplet not displayed by *S*.

Let *A*=LEAF_*S*_(*u*_*A*_) and *B*=LEAF_*S*_(*u*_*B*_) where *u*_*A*_ and *u*_*B*_ are the children of ROOT(*S*). Note that *A* and *B* partition the set of species *Σ*. As *H*_*G*,*s*_ is connected, there exists *a*∈*A*,*b*∈*B* such that *a* *b*∈*F*. Therefore there exist *x*,*y*∈*V* such that *x*,*y* are in the same connected component *C* of *G*(*∅*),*s*(*x*)=*a*,*s*(*y*)=*b* and *x* *y*∉*M*∪*U*.

As *G* ^′^[*C*] is connected, there exists a chordless path *P* from *x* to *y* in *G* ^′^. By Proposition 2, *G* ^′^ is *P*_4_-free. This implies that *P* contains, in addition to *x* and *y*, a third vertex *z* such that *x* *z*∈*E* ^′^ and *z* *y*∈*E* ^′^.

Assume without loss of generality that *s*(*z*)∈*A* (the case *s*(*z*)∈*B* is symmetric). Then we have *s*(*x*)*s*(*y*)|*s*(*z*)∈*P*_3_(*G* ^′^). Note however that LCA_*S*_(*s*(*y*),*s*(*z*))=ROOT(*S*) (as *s*(*z*)∈*A*,*s*(*y*)∈*B*), while LCA_*S*_(*s*(*x*),*s*(*z*)) is a descendant of LCA_*S*_(*A*). It follows that LCA_*S*_(*s*(*x*),*s*(*z*)) is different from LCA_*S*_(*s*(*y*),*s*(*z*)), and so *s*(*x*)*s*(*y*)|*s*(*z*) is not displayed by *S*. □

The next lemma shows how to use connected components of the species graph in order to freeze some unknown edges to orthology relations between genes.

#### **Lemma 3**

Let (*G*,*s*) be a constraint graph reduced by Reduction Rule 1 such that the species graph *H*_*G*,*s*_ is not connected.

Let *A* be the vertices of a connected component of the species graph *H*_*G*,*s*_ and let *B*=*Σ*∖*A*. Let *G*_*A*_=(*V*_*A*_,*M*[*V*_*A*_]⊎*U*[*V*_*A*_]) and *G*_*B*_(*V*_*B*_,*M*[*V*_*B*_]⊎*U*[*V*_*B*_]), where *V*_*A*_=*s* ^−1^(*A*) and *V*_*B*_=*s* ^−1^(*B*). There exists a consistent sandwich graph of (*G*,*s*) if and only if there exist consistent sandwich graphs of $\phantom {\dot {i}\!}(G_{A},s_{|V_{A}})$ and of $(G_{B},s_{|V_{B}})\phantom {\dot {i}\!}$.

#### *Proof*

Let $G^{\prime }_{A}$ and $G^{\prime }_{B}$ be respectively consistent sandwich graphs of $\phantom {\dot {i}\!}(G_{A},s_{|V_{A}})$ and of $\phantom {\dot {i}\!}(G_{B},s_{|V_{B}})$. Suppose that $G^{\prime }_{A}$ is consistent with the species tree *S*_*A*_ and $G^{\prime }_{B}$ with *S*_*B*_. For every connected component *C* of *G*(*∅*), let $G^{\prime }_{C}$ be the series composition of $G^{\prime }_{A}[C]$ and $G^{\prime }_{B}[C]$ and let *G* ^′^=(*V*,*E* ^′^) be the disjoint union of all $G^{\prime }_{C}$’s. We now show that (*G* ^′^,*s*) is a consistent sandwich graph of (*G*,*s*).

As $G_{A}^{\prime }$ and $G_{B}^{\prime }$ are cographs, by construction *G* ^′^ is a cograph too. Now, as $G_{A}^{\prime }$ and $G_{B}^{\prime }$ are respectively sandwich graphs of $\phantom {\dot {i}\!}(G_{A},s_{|V_{A}})$ and $(G_{B},s_{|V_{B}})\phantom {\dot {i}\!}$, and as there is no edge in *M* between different connected components of *G*(*∅*), we have that *M*⊆*E* ^′^. By construction of *H*_*G*,*s*_ and the fact that *H*_*G*,*s*_ has no edges between *A* and *B*, for every connected component *C* of *G*(*∅*), if *x*∈*V*_*A*_∩*C* and *y*∈*V*_*B*_∩*C*, then *x* *y*∈*M*∪*U*. As $G_{A}^{\prime }$ and $G_{B}^{\prime }$ are respectively sandwich graphs of $(G_{A},s_{|V_{A}})\phantom {\dot {i}\!}$ and $(G_{B},s_{|V_{B}})\phantom {\dot {i}\!}$, this implies that *E* ^′^⊆*M*∪*U*. It follows that *G* ^′^ is a sandwich graph of *G*.

Now consider the species tree *S* obtained from *S*_*A*_ and *S*_*B*_ by adding a root whose children are ROOT(*S*_*A*_) and ROOT(*S*_*B*_). We claim that (*G* ^′^,*s*) is consistent with *S*. Consider a triplet *s*(*x*)*s*(*y*)|*s*(*z*)∈*P*_3_(*G* ^′^,*s*). We distinguish two cases:

- If {*s*(*x*),*s*(*y*),*s*(*z*)}⊆*A* (the case {*s*(*x*),*s*(*y*),*s*(*z*)}⊆*B* is symmetric), then *s*(*x*)*s*(*y*)|*s*(*z*)∈*P*_3_(*G*_*A*_) and is displayed by *S*_*A*_ and thereby by *S* as well.
- Otherwise, as *x* *z*,*y* *z*∈*E* ^′^,*x* and *y* are connected in *G* ^′^ and so by construction of *G* ^′^, we have that *x*,*y*∈*C* for some connected component *C* of *G*(*∅*). As *x* *y*∉*E* ^′^, by construction of $G_{C}^{\prime }$ either {*s*(*x*),*s*(*y*)}⊆*A* or {*s*(*x*),*s*(*y*)}⊆*B*. Suppose w.l.o.g that the former holds, implying *s*(*z*)∈*B*. Observe then that *s*(*x*)*s*(*y*)|*s*(*z*) is displayed by *S*. Indeed, we have lca_*S*_(*s*(*x*),*s*(*z*))=lca_*S*_(*s*(*y*),*s*(*z*))=root(*S*), and lca_*S*_(*s*(*x*)*s*(*y*)) is a descendant of root(*S*_*A*_).

The converse follows from Corollary 1. □

The correctness of the next branching rule follows from Lemma’s 2 and 3.

#### **Branching Rule 1**

Let (*G*,*s*) be a constraint graph reduced by Reduction Rule 1 such that the species graph *H*_*G*,*s*_ is not connected. Let *A* be a connected component of the species graph *H*_*G*,*s*_ and let *B*=*Σ*∖*A*. Solve CONSISTENT SANDWICH SUBGRAPH on $(G_{A}, s_{|V_{A}})\phantom {\dot {i}\!}$ and $(G_{B}, s_{|V_{B}})\phantom {\dot {i}\!}$ where *V*_*A*_=*s* ^−1^(*A*) and *V*_*B*_=*s* ^−1^(*B*). If there exist $G^{\prime }_{A}=(V_{A},E^{\prime }_{A})$ and $G^{\prime }_{B}=(V_{B},E^{\prime }_{B})$ that are respectively consistent sandwich graphs of $(G_{A}, s_{|V_{A}})\phantom {\dot {i}\!}$ and $(G_{B}, s_{|V_{B}})\phantom {\dot {i}\!}$, then return $G^{\prime }=(V,E^{\prime }_{A}\cup E^{\prime }_{B}\cup M^{\prime })$, where *M* ^′^={*x* *y*∈*M*∪*U*:*x*∈*V*_*A*_,*y*∈*V*_*B*_}. Otherwise, return NULL.

**Fig. 2:**
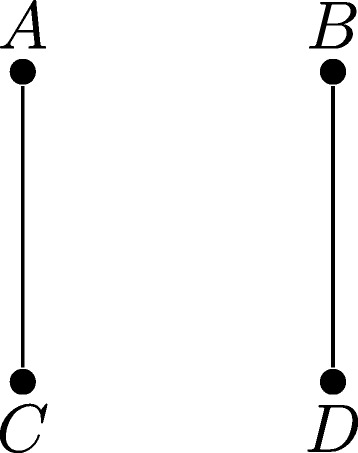
Example of the species graph *H*_*G*,*s*_ derived from (*G*,*s*) after an application of Reduction Rule 1. The edge *AC* is due to non-edge *a*_1_ *c*_1_; edge *BD* is due to non-edges *b*_1_ *d*_1_ and *b*_2_ *d*_2_. As *H*_*G*,*s*_ is not connected, we can apply Branching Rule 1

Consider again the example of Fig. 1, after the unknown edge *d*_1_ *a*_2_ has been removed by Reduction Rule 1. Because one connected component has non-edges *a*_1_ *c*_1_,*b*_1_ *d*_1_ and the other has non-edge *b*_2_ *d*_2_, the edges in *H*_*G*,*s*_ will be *AC* and *BD* (see Fig. 2). Thus, Branching Rule 1 will split the constraint graph into two parts, one restricted to *a*_1_,*c*_1_,*a*_2_,*c*_2_, and one restricted to *b*_1_,*d*_1_,*b*_2_,*d*_2_.

We can now give the pseudocode of the algorithm, which essentially consists of alternately applying Reduction Rule 1 and Branching Rule 1.



#### **Theorem 2**

Given a constraint graph (*G*,*s*), the CONSISTENT ORTHOLOGY GRAPH SANDWICH problem can be solved in *O*(*n* ^3^) time, where *n* is the number of genes in *G*.

#### *Proof*

The correctness of Algorithm 1 follows from the correctness of Reduction Rule 1 (Lemma 1) and Branching Rule 1 (Lemma’s 2 and 3).

To analyze the running time of Algorithm 1, we simply observe that the recursive calls define a binary tree structure with at most *O*(|*Σ*|)=0(*n*) nodes. As each step of the recursion can clearly be performed in quadratic time, so the complexity follows. □

We can adapt the algorithm to cases when the species tree *S* is partially known, by adjusting the construction of *H*_*G*,*s*_. In particular, for any *x*,*y*,*z*∈*V* for which it is known that *S* displays the triplet *s*(*x*)*s*(*y*)|*s*(*z*), we add *s*(*x*)*s*(*y*) as an edge in *H*_*G*,*s*_.

Algorithm 1 has important applications. When the species tree is not known, it allows us to differentiate constraint graphs that are consistent with a species tree from those that are not; the latter cannot be depicted by a consistent DS-tree, and should be considered as phylogenetically irrelevant and discarded. When the species tree *S* is known and a given constraint graph *C* is not consistent with it, the sandwich graph returned by Algorithm 1 shows to what extent *C* and *S* are in contradiction. Furthermore if *S* contains some uncertainties, it allows us to see if the contradictions between *C* and *S* lie in the “uncertainty zone” of *S*. This may help to correct the species tree.

**Fig. 3:**
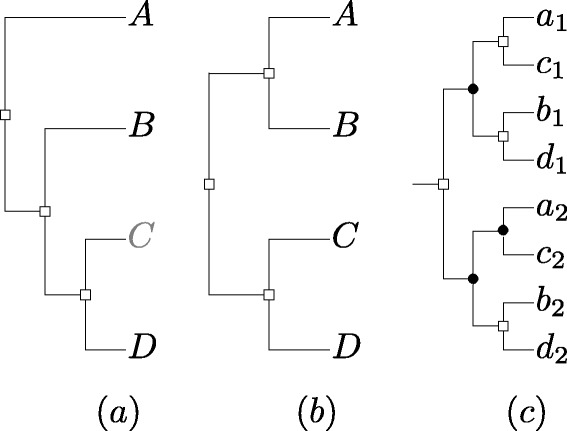
Example of (**a**) a species tree where the placement of *C* is uncertain, and **b**) another species tree that can be derived from the first by changing the position of *C*. The DS-tree in (**c**) is not consistent with the species tree in (**a**)(assuming *s*(*a*_1_) = *s*(*a*_2_) = *A*,*s*(*b*_1_) = *s*(*b*_2_) = *B*,*s*(*c*_1_) = *s*(*c*_2_)=*C*,*s*(*d*_1_)=*s*(*d*_2_)=*D*), but it is consistent with the species tree in (**b**). In (**c**), *circles* represent speciation events, and *squares* represent duplication events

As an example of the last appplication, suppose that we have the species tree given in Fig. 3(a), but the relative position of species *C* in this tree is uncertain. Suppose in addition we are given the constraint graph (*G*,*s*) given in Fig. 1. The DS-tree in Fig. 3(c) is a DS-tree for (*G*,*s*), but is not consistent with the Fig. 3(a). However, it is consistent with the species tree in Fig. 3(b), which can be derived from Fig. 3(a) by moving species *C*.

See the “Results and Discussion” section for an example of application on real data.

### Hardness of optimizing the duplication nodes

Given a constraint graph (*G*,*s*) for which there exist several possible DS-trees, we may be interested in finding one minimizing the number of duplication nodes. Duplication minimization is a well-known criterion in phylogenomics [4,11]; for example, it is used to resolve polytomies in gene trees in [19] and to estimate the species tree in [20].

In this section, we consider the following three optimization variants of the ORTHOLOGY GRAPH SANDWICH problem in which the number of duplication nodes has to be minimized. We prove hardness results for each of these problems.

*k*-DUPLICATION ORTHOLOGY GRAPH SANDWICH problem (*k*-DOGS)

**Input:** a constraint graph (*G*,*s*) and an integer *k*;

**Output:** does there exists a DS-tree (*T*,*s*) containing at most *k* duplication nodes, whose orthology graph is a sandwich of *G*?

The above problem is equivalent to asking if (*G*,*s*) is satisfiable and there exists a DS-tree for (*G*,*s*) containing at most *k* duplication nodes.

SPECIES TREE CONSISTENT *k*-DUPLICATION ORTHOLOGY GRAPH SANDWICH problem (S-CONS- *k*-DOGS)

**Input**: a constraint graph (*G*,*s*), with *G*=(*V*,*M*⊎*U*) and *s*:*V*→*Σ* a species assignment, a species tree *S* on *Σ* and an integer *k*;

**Question**: does there exist a DS-tree (*T*,*s*) containing at most *k* duplication nodes, whose orthology graph is a sandwich of *G*, and is consistent with *S*?

CONSISTENT *k*-DUPLICATION ORTHOLOGY GRAPH SANDWICH Problem (CONS- *k*-DOGS)

**Input**: a constraint graph (*G*,*s*), with *G*=(*V*,*M*⊎*U*) and *s*:*V*→*Σ* a species assignment, and an integer *k*;

**Question**: does there exist a DS-tree (*T*,*s*) containing at most *k* duplication nodes and a species tree *S*, such that the orthology graph of (*T*,*s*) is a sandwich of *G* and is consistent with *S*?

We first provide a reduction from 3-COLORING that proves that *k*-DOGS is para-NP-hard [21] with respect to the number of duplication nodes *k* (that is, *k*-DOGS is NP-hard for some fixed *k*). This implies that *k*-DOGS does not belong to the complexity class **X** **P**, meaning that the problem cannot be solved in time *O*(*n* ^*f*(*k*)^) for some function *f*(.). In what follows, [*k*] denotes the set {1,⋯,*k*}.

*k*-COLORING Problem

**Input**: a (connected) graph *G*=(*V*,*E*);

**Question**: does there exist a *k*-coloring *c*:*V*→[*k*] such that for every *x* *y*∈*E*, *c*(*x*)≠*c*(*y*)?

The following lemma will be useful in this section. An equivalent version of this lemma could be written in terms of cographs, and we believe a proof for such a lemma should already exist in the literature. However, as we were unable to find such a proof, we give one here.

#### **Lemma 4**

Let (*G*,*s*) be an orthology graph with a DS-tree containing at most *k* duplication nodes. Then we can find a *k*+1 coloring of its complement $\overline {G}$ in polynomial time.

#### *Proof*

Let (*G*=(*V*,*E*),*s*) be an orthology graph. We prove the claim by induction on |*V*|.

If |*V*|=1, then there are 0 duplication nodes in a DS-tree for (*G*,*s*), and $\overline {G}$ has a 1-coloring, as required.

So now suppose the claim holds for all orthology graphs (*G* ^′^=(*V* ^′^,*E* ^′^),*s* ^′^) with |*V* ^′^|<|*V*|. Let (*T*,*σ*) be a DS-tree for (*G*,*s*) with at most *k* duplication nodes. Consider ROOT(*T*). If ROOT(*T*) is a duplication node, then *G* is disconnected, and we can find a partition *V*=*V*_*A*_⊎*V*_*B*_ such that there is no edge between *V*_*A*_ and *V*_*B*_ in *G*. Moreover, the number of duplication nodes in *T* is *k*_*A*_+*k*_*B*_+1, where *k*_*A*_ is the number of duplication nodes in a DS-tree for *G*[*V*_*A*_], and *k*_*B*_ is the number of duplication nodes in a DS-tree for *G*[*V*_*B*_]. By the inductive hypothesis, there exists a *k*_*A*_+1 coloring for $\overline {G[V_{A}]}$, and a *k*_*B*_+1 coloring for $\overline {G[V_{B}]}$. It is clear that we can combine these colorings into a *k*_*A*_+1+*k*_*B*_+1≤*k*+1 coloring of $\overline {G}$.

If ROOT(*T*) is a speciation node, then $\overline {G}$ is disconnected, and we can find a partition *V*=*V*_*A*_⊎*V*_*B*_ such that there are no edges between *V*_*A*_ and *V*_*B*_ in $\overline {G}$. Moreover, the number of duplication nodes in a DS-tree for *G*[*V*_*A*_] (*G*[*V*_*B*_], respectively) is at most *k*. By the inductive hypothesis, there exists a *k*+1 coloring for $\overline {G[V_{A}]}$ and a *k*+1 coloring for $\overline {G[B]}$ and these can be combined into a *k*+1 coloring for *G*.

This proof can be turned into a polynomial time algorithm as follows. If *G* is disconnected, find a partition *V*=*V*_*A*_⊎*V*_*B*_ with no edges between *V*_*A*_ and *V*_*B*_ in *G*, and recursively find colorings for $\overline {G[V_{A}]}$ and $\overline {G[V_{B}]}$, adjusting the coloring on $\overline {G[V_{B}]}$ to assign different values from those assigned by the coloring on $\overline {G[V_{A}]}$.

Otherwise, find a partition *V*=*V*_*A*_⊎*V*_*B*_ with no edges between *V*_*A*_ and *V*_*B*_ in $\overline {G}$, and recursively find colorings for $\overline {G[V_{A}]}$ and $\overline {G[V_{B}]}$. As each recursion splits the set of vertices and each recursive step takes polynomial time, the whole algorithm takes polynomial time. □

#### **Lemma 5**

Given a connected graph *G*=(*V*,*E*), define a constraint graph (*H*=(*V*,*M*⊎*U*),*s*) by setting *M*=*∅* and $U=\overline E$, and letting *s*:*V*→*Σ* be an arbitrary species assignment such that each gene in *V* is assigned to a different species. Then for any integer *k*>0,*G* is *k*-colorable if and only if (*H*,*s*) has a solution with at most *k*−1 duplication nodes. Furthermore if such a solution exists then there exists a solution consistent with an arbitrary species tree on *Σ*.

#### *Proof*

Assume that *G* is *k*-colorable, with *c*:*V*→[*k*] a *k*-coloring of *G*. Let *V*_*i*_=*c* ^−1^(*i*) for each *i*∈[*k*]. Thus *V*_1_,…*V*_*k*_ form a partition of *V*. For each *i*∈[*k*], let $(T_{i}, s_{|V_{i}})\phantom {\dot {i}\!}$ be an arbitrary DS-tree with leaves *V*_*i*_ such that every internal node is a speciation node, and let *x*_*i*_ denote the root of *T*_*i*_. We now construct a DS-tree (*T*,*s*) as follows. Let *z*_1_,…,*z*_*k*−1_ be duplication nodes such that ROOT(*T*)=*z*_1_, such that for each *i*∈[*k*−2],*z*_*i*_ has child nodes *x*_*i*_ and *z*_*i*+1_, and the children of *z*_*k*−1_ are *x*_*k*−1_ and *x*_*k*_. Now consider the graph *H* ^′^=(*V*,*E* ^′^) obtained from the disjoint union of cliques on *V*_*i*_ for 1≤*i*≤*k*. Observe that *H* ^′^ is a sandwich graph of (*H*,*s*). Moreover by construction, we have that *x* *y*∈*E* ^′^ if and only if LCA_*T*_(*x*,*y*) is a speciation node. Moreover (*T*,*s*) has *k*−1 duplication nodes, so *H* ^′^ is a solution. To conclude the proof of the first claim, observe that the converse follows from Lemma 4. To see the second claim, observe that as *H* ^′^ is a disjoint union of cliques, *P*_3_(*H* ^′^,*s*)=*∅* and therefore (*H* ^′^,*s*) is consistent with any species tree on *Σ*. □

**Fig. 4:**
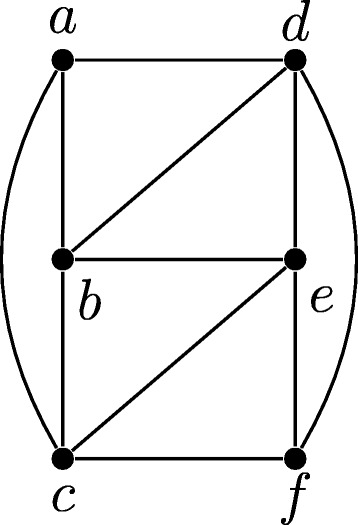
A graph *G*=(*V*,*E*)

**Fig. 5:**
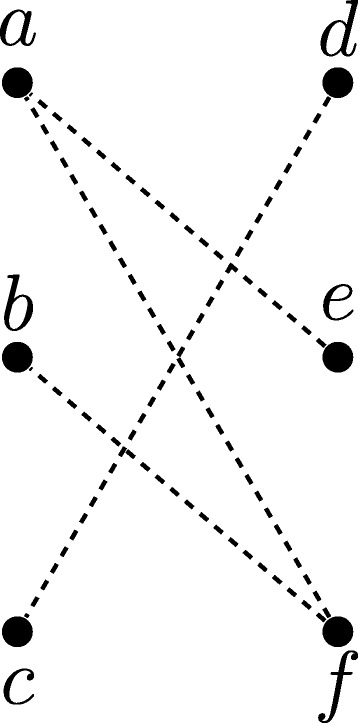
A constraint graph (*H*=(*V*,*M*⊎*U*),*s*) derived from *G* by setting *M*=*∅* and $U=\overline E$, and letting *s*:*V*→*Σ* be an arbitrary species assignment such that each gene is mapped to a different species

**Fig. 6:**
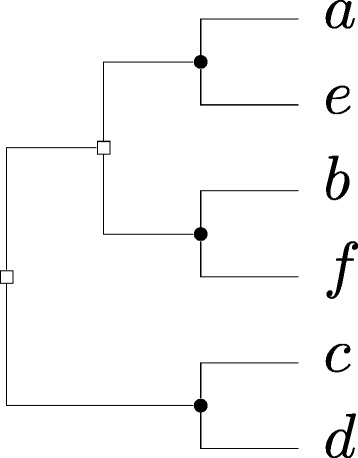
A DS-tree for (*H*=(*V*,*M*⊎*U*),*s*). Note that the partition {*a*,*e*},{*b*,*f*},{*c*,*d*} corresponds to a 3-coloring of *G*. *Circles* represent speciation events, and *squares* represent duplication events

As an example of the construction in the proof above, consider the graph *G*=(*V*,*E*) given in Fig. 4. The corresponding constraint graph (*H*=(*V*,*M*⊎*U*),*s*) is given in Fig. 5, and a DS-tree for this constraint graph is given in Fig. 6. As this DS-tree has 2 duplication nodes, *G* has a 3-coloring. In particular, following the structure of Fig. 6, we observe that there is a 1-coloring of *G*[{*a*,*e*}] (as these vertices are not adjacent in *G*), and a 1-coloring of *G*[{*b*,*f*}]. Combining these colorings gives a 2-coloring of *G*[{*a*,*b*,*e*,*f*}], which can then be combined with a 1-coloring of *G*[{*c*,*d*}] to give a 3-coloring of *G*.

We now prove the NP-hardness of 2-DOGS, S-CONS-2-DOGS or CONS-2-DOGS, using Lemma 5 and the fact that 3-COLORING is NP-hard [22].

#### **Theorem 3**

2-DOGS is NP-hard.

#### *Proof*

Given an instance *G*=(*V*,*E*) of 3-COLORING, let (*H*,*s*) be the constraint graph given by Lemma 5. Then by Lemma 5, (*H*,*s*,2) is a YES-instance of *k*-DOGS if and only if *G* is 3-colorable. As 3-COLORING is NP-hard, so is 2-DOGS. □

Using the same technique as for Theorem 3, we can prove the same NP-hardness result for S-CONS-2-DOGS and CONS-2-DOGS. The proofs are identical to that of Theorem 3, except that in the case of Theorem 4 we construct an arbitrary species tree *S* on *Σ* in addition to the constraint graph (*H*,*s*).

#### **Theorem 4**

S-CONS-2-DOGS is NP-hard.

#### **Theorem 5**

CONS-2-DOGS is NP-hard.

Let MINDOGS, S-CONS-MINDOGS, and CONS-MINDOGS denote the minimization versions of *k*-DOGS, S-CONS- *k*-DOGS, and CONS- *k*-DOGS respectively, in which we want to find a solution with the minimum number of duplication nodes. Let GRAPH COLORING denote the minimization version of *k*-COLORING. As GRAPH COLORING has no polynomial time $n^{1-\epsilon ^{\prime }}$-approximation for any *ε* ^′^>0, unless P=NP [23], we can prove the following theorem.

#### **Theorem 6**

For any *ε*>0, there is no polynomial time algorithm that takes as input an instance of MINDOGS, and returns a solution with at most *n* ^1−*ε*^·*k* duplication nodes if there exists a solution with at most *k* duplication nodes, unless *P*=*N* *P*.

#### *Proof*

Let *G*=(*V*,*E*) be an instance of GRAPH COLORING. Without loss of generality we may assume that *G* is connected. Let (*H*,*s*) be the constraint graph given by Lemma 5.

Now for any *ε*>0, fix an integer *n*_0_ and *ε* ^′^>0 such that $ n^{1-\epsilon } +1 < n^{1-\epsilon ^{\prime }}$ for any *n*≥*n*_0_.

Suppose that there exists a polynomial-time *n* ^1−*ε*^-approximation for MINDOGS, i.e. an algorithm that for any instance (*H*,*s*) with *n* vertices, finds a solution with at most *n* ^1−*ε*^·*k* duplication nodes if there exists a solution with at most *k* duplication nodes. We show that there exists a polynomial-time $n^{1-\epsilon ^{\prime }}$-approximation for GRAPH COLORING.

Let *G* be an instance of GRAPH COLORING with *n* vertices, and suppose without loss of generality that *n*≥*n*_0_ (as otherwise the problem can be solved exactly in polynomial time). Let (*H*,*s*) be the instance of MINDOGS constructed from *G* as above. Now run the supposed approximation algorithm for MINDOGS on (*H*,*s*). If *G* is *k*-colorable for any *k*>1, then by Lemma 5, there exists a solution for (*H*,*s*) with at most *k*−1 duplication nodes. Therefore if *G* is *k*-colorable, the algorithm returns a solution with at most *n* ^1−*ε*^·(*k*−1) duplication nodes. (Note that we may assume the solution contains at least 1 duplication node, as otherwise *G* would be disconnected). Let (*H* ^′^,*s*) be the orthology graph for this solution. Then by Lemma 4, we have a *n* ^1−*ε*^·(*k*−1)+1-coloring for $\overline {H^{\prime }}$. As *G* is a subgraph of $\overline {H^{\prime }}$, this is also a *n* ^1−*ε*^·(*k*−1)+1-coloring for *G*.

As $n \ge n_{0}, n^{1-\epsilon }\cdot (k-1) +1 \le (n^{1-\epsilon } +1) \cdot k \le n^{1-\epsilon ^{\prime }}\cdot k$ and so we have a polynomial time $n^{1-\epsilon ^{\prime }}$-approximation for GRAPH COLORING, a contradiction. □

Using the same technique as for Theorem 6, we can prove the same inapproximability result for S-CONS-MINDOGS and CONS-MINDOGS. The proofs are identical to that of Theorem 6, except that in the case of Theorem 7 we construct an arbitrary species tree *S* on *Σ* in addition to the constraint graph (*H*,*s*).

#### **Theorem 7**

For any *ε*>0, there is no polynomial time algorithm that takes as input an instance of S-CONS-MINDOGS, and returns a solution with at most *n* ^1−*ε*^·*k* duplication nodes if there exists a solution with at most *k* duplication nodes, unless *P*=*N* *P*.

#### **Theorem 8**

For any *ε*>0, there is no polynomial time algorithm that takes as input an instance of CONS-MINDOGS, and returns a solution with at most *n* ^1−*ε*^·*k* duplication nodes if there exists a solution with at most *k* duplication nodes, unless *P*=*N* *P*.

To summarise the results in this section: given a constraint graph on *n* vertices, it is NP-hard to find a DS-tree for that graph with at most *k* duplication nodes, even when *k*=2. This holds regardless of whether we require the DS-tree to be consistent, or whether we are given a species tree that it should be consistent with. Viewed as a minimization problem, it is NP-hard even to find an *n* ^1−*ε*^-approximate solution, for any *ε*>0.

## Results and Discussion

We integrated Algorithm 1 to the software provided at [24] by the authors of [9]. Note that the previous version of the program only permitted to check satisfiability and consistency of a constraint graph with respected to a given species tree *S*.

We used the modified software to reanalyze the data set in [9]. This data set was constructed by randomly choosing 265 gene families of vertebrates with more than 20 genes from *Ensembl* [25]. Each gene family was then analysed with ProteinOrtho [26] using 9 different parameter settings, yielding 2385 different constraint graphs. Here *S* is the Ensembl species tree, which can be downloaded at [27].

For this data set we have that, apart from one case, all satisfiable constraint graphs are also consistent. In 533 out of 2385 cases, the constraint graph was found to be consistent, but not consistent with *S*. We were interested in finding out how greatly the graphs in this set (denoted $\mathcal {CG}$) conflicted with *S*. Indeed, some nodes in the Ensembl species tree, for example the position of *Equus*, *Tupaia* and *Cavia*, do not enjoy a consensus in the community, so some contradictions with *S* are expected.

Note that we can use the graph *G* ^′^ outputted by Algorithm 1 to obtain a species tree in the following way: we compute the set $\mathcal {T}$ of all *P*_3_(*G* ^′^,*s*) and then feed $\mathcal {T}$ to the BUILD algorithm [18], which will return a species tree displaying all the triplets in $\mathcal {T}$ (in practice, our implementation of Algorithm 1 is able to construct a species tree directly).

This species tree can fail to be binary, if the information contained in $\mathcal {T}$ is sparse (this is actually the case for our data set: the maximum number of internal nodes over all species trees reconstructed by our approach from constraint graphs in $\mathcal {CG}$ was 6, with an average of 1.5).

**Fig. 7:**
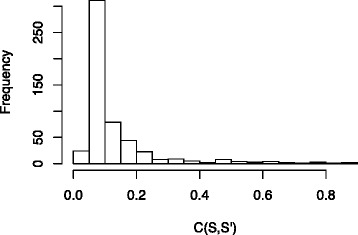
The histogram of the values of *c*(*S*,*S* ^′^) for our data set

To estimate the discordancy between the Ensembl species tree *S* and each of the species trees *S* ^′^ reconstructed by our approach for a constraint graph in $\mathcal {CG}$, we did the following: for each pair (*S*,*S* ^′^) we constructed a tree *S* ^′′^ displaying the maximum number of triplets of *S* not contradicting *S* ^′^ using PhySIC_IST [28]. We then computed the number of triplets displayed by *S* not in *S* ^′′^, as a proportion of the total number of triplets displayed by *S*: the higher this number is, the higher the conflict between *S* and *S* ^′^. This number, denoted *c*(*S*,*S* ^′^), can be used to differentiate gene families that are good markers (i.e. markers highly coherent with the given species tree, which will have a low *c*(*S*,*S* ^′^)) from gene families that are bad markers (with a high *c*(*S*,*S* ^′^)). The histogram of the values of *c*(*S*,*S* ^′^) for our data set is given in Fig. 7. This shows that several constraint graphs, even though not consistent with *S*, are not in high contradiction with it and thus the corresponding gene families can still be considered as good markers.

## Conclusions

In this paper, we extend the results of [9] by giving a *O*(*n* ^3^) time algorithm to decide whether $\mathcal {C}$ is consistent, even when the species tree is not known and $\mathcal {C}$ is not full. We also incorporated this algorithm into the software provided at [24]. The algorithm has important applications in providing evidence for the structure of a species tree when that species tree is unknown. It also allows us to see how much an ‘inconsistent’ set of constraints is in conflict with a known species tree, as the algorithm returns a species tree for which those constraints are consistent, if any exists. On the negative side, we show that the problem of minimizing duplications nodes in DS-trees is NP-hard even when the number of duplications is very small, and it is also hard to find approximate solutions for this criterion.
